# A Network Model of Health-Related Changes after a Lifestyle-Enhancing Treatment in Patients with Severe Mental Illness: the MULTI Study VI^☆^

**DOI:** 10.1016/j.ijchp.2024.100436

**Published:** 2024-01-09

**Authors:** Lydia Pieters, Tessa Blanken, Kirsten van Lunteren, Peter van Harten, Jeroen Deenik

**Affiliations:** a Research Department, Psychiatric Centre GGz Centraal, Amersfoort, The Netherlands; b Department of Mental Health and Neuroscience, Maastricht University, Maastricht, The Netherlands; c Department of Psychological Methods, University of Amsterdam, Amsterdam, The Netherlands; d Department of Clinical Psychology, University of Amsterdam, Amsterdam, The Netherlands

**Keywords:** Schizophrenia, physical activity, physical health, quality of life, psychosocial functioning

## Abstract

**Background/Objective:**

The effects of lifestyle interventions on physical and mental health in people with severe mental illness (SMI) are promising, but its underlying mechanisms remain unsolved. This study aims to examine changes in health-related outcomes after a lifestyle intervention, distinguishing between direct and indirect effects.

**Method:**

We applied network intervention analysis on data from the 18-month cohort Multidisciplinary Lifestyle enhancing Treatment for Inpatients with SMI (MULTI) study in 106 subjects (62% male, mean age=54.7 (SD=10.8)) that evaluated changes in actigraphy-measured physical activity, metabolic health, psychopathology, psychosocial functioning, quality of life and medication use after MULTI (n=65) compared to treatment as usual (n=41).

**Results:**

MULTI is directly connected to decreased negative symptoms and psychotropic medication dosage, and improved physical activity and psychosocial functioning, suggesting a unique and direct association between MULTI and the different outcome domains. Secondly, we identified associations between outcomes within the same domain (e.g., metabolic health) and between the domains (e.g., metabolic health and social functioning), suggesting potential indirect effects of MULTI.

**Conclusions:**

This novel network approach shows that MULTI has direct and indirect associations with various health-related outcomes. These insights contribute to the development of effective treatment strategies in people with severe mental illness.

## Introduction

Improving the poor physical health of people with severe mental illness (SMI) is a major challenge in psychiatry (Firth et al., 2019). People with SMI have a substantially reduced life expectancy of 15-20 years compared to the general population (Hjorthøj et al., 2017; Laursen et al., 2019), largely caused by poor cardiometabolic health (Correll et al., 2022; Walker et al., 2015). Modifiable lifestyle factors such as reduced physical activity and increased sedentary behavior (Kruisdijk et al., 2017; Stubbs et al., 2017; Stubbs et al., 2016a; Stubbs et al., 2016b; Vancampfort et al., 2013), smoking (de Leon & Diaz, 2005), and poor nutrition (Teasdale et al., 2017) play a major role in these poor health outcomes. Furthermore, people with SMI experience difficulties in psychosocial functioning and report a decreased quality of life, which can be related to various factors such as psychopathological symptoms, social and occupational impairments, cognitive deficits, impairments in emotional experience, and self-stigma (Sarraf et al. 2022; Świtaj et al., 2012). Studies have related various lifestyle factors to these psychosocial impairments, including physical activity, sedentary behavior, and daily activities such as problematic smartphone use and internet gaming (K.C. Chang et al., 2022; Y.H. Chang et al., 2020; Deenik et al., 2018a; Firth et al., 2019).

Over the past decades, research has aimed to improve this health disparity by developing targeted interventions aimed at lifestyle factors in people with SMI (Chu et al., 2018; Firth et al., 2019, 2020; Huang et al., 2022; Teasdale et al., 2017). Systematic reviews and meta-analyses have demonstrated the efficacy of physical activity interventions for cardiometabolic health, psychiatric symptoms, quality of life, and global and cognitive functioning (Firth et al., 2019, 2020). Dietary interventions, often combined with physical activity interventions and psycho-education, yielded improvements in parameters of the metabolic syndrome and cardiorespiratory fitness (Firth et al., 2020; Teasdale et al., 2017).

Although there is emerging evidence showing the efficacy of lifestyle interventions in people with SMI, evidence on its effectiveness in daily routine healthcare is currently limited (Deenik, 2019; Jakobsen et al., 2017; Naslund et al., 2017; Stubbs et al., 2018). There is a need for more research in real-world settings, including integrated lifestyle interventions and measuring and relating improvements on different physical and mental health-related outcome domains. Understanding of the mechanisms behind lifestyle improvement and how lifestyle interventions relate to different cardiometabolic health, psychosocial functioning, and mental health is essential for development of effective lifestyle intervention strategies in people with SMI. The relationship between lifestyle factors, cardiometabolic health parameters, mental health, social functioning and quality of life is complex and involves reciprocal interactions (Firth et al., 2020; Vancampfort et al., 2012).

A possible way of gaining insight into the interplay between these multiple factors is offered by the network approach, in which the organization of a system is studied by identifying system components (nodes) and the relations among them (edges) (Borsboom et al., 2021). More recently, network intervention analysis (NIA) has been introduced as an extension of network models to identify treatment-induced changes in symptoms and their association structure over time (Blanken et al., 2019). In this study, we applied NIA to examine direct and indirect changes in health-related outcomes (i.e., metabolic health, psychiatric symptom severity, social functioning, quality of life, and medication use) after a multidisciplinary lifestyle intervention in patients with SMI. To this end, we used data from the MULTI study, an 18-month cohort study evaluating a multidisciplinary lifestyle-enhancing treatment for inpatients with SMI (Deenik et al., 2019; Deenik et al., 2018a; Deenik et al., 2018b). Data from this study has been previously analyzed and showed significant improvements in physical activity, metabolic health, and psychosocial functioning (Deenik et al., 2019; Deenik et al., 2018a) and a decreased use of psychotropic medication (Deenik et al., 2018b) after 18-months of MULTI compared to treatment as usual (TAU). In the present study, we applied NIA to gain insight in the direct and indirect changes in health-related outcomes.

## Methods

### Study Design

Data was used from the MULTI study; a cohort study evaluating a multidisciplinary lifestyle-enhancing treatment for inpatients with SMI. MULTI was implemented in February 2014 at wards for long-term mental healthcare (i.e., ≥1 year hospitalization) of a mental health care institution in the Netherlands (GGz Centraal). Due to the observational nature of the study, whereby MULTI was already implemented pragmatically at three wards before the start of this study, no randomization took place. For that reason, we accounted for the baseline variables that differed significantly between groups in the analysis, in line with previous publications of this study (Deenik et al., 2018a, 2018b). Full details on the study protocol are reported elsewhere (Deenik et al., 2019). The study protocol was approved by Medical Ethical Committee of the Isala Academy (case 14.0678). All subjects gave written informed consent in accordance with the Declaration of Helsinki.

### Study Population

The sample consisted of subjects with SMI who had been hospitalized for at least one year at one of the inpatient wards. They were included if they had not received any other intervention related to lifestyle within the 18 months since the start of MULTI. They were excluded if they did not understand the content of MULTI, in consultation with their attending psychiatrist. For the current analysis, we included the baseline and follow-up data of subjects with sufficient actigraphy data (a wear time of ≥6 hours/day for ≥3 days (Deenik et al., 2017)) and available data on physical health and social functioning outcome measures (n=106; n=65 for MULTI and n=41 for TAU).

### Intervention

MULTI is a multidisciplinary lifestyle-enhancing treatment that focused on decreasing sedentary behavior, increasing physical activity, and improving dietary habits to achieve overall lifestyle change. The treatment method was based on improving the daily structure and participating in an active day program, including sports- and work-related activities, psychoeducation, and daily living skills training. The frequency, intensity and the kind of activities could vary between patients and wards, as they were tailored to the individual patient's illness severity, capabilities, and interests. Participation of the ward nursing team a core element of MULTI, which contributed to the culture change and support of patients. Full details on the intervention program are reported elsewhere and are provided in the supplement (Deenik et al., 2019).

Patients who received TAU continued their treatment at their wards, which mainly concerned pharmacological treatment and a less structured day program that did not include any supported lifestyle interventions or adjustments.

### Assessments

At baseline, severity of psychopathology was evaluated using the Clinical Global Impression (CGI) severity index (Nolen, 1990), consisting of one item (global severity of disease), rated by the psychiatrist from 1 (not at all ill) to 7 (extremely ill). At baseline and 18-month follow-up data on psychopathology, psychosocial functioning, quality of life, physical health, medication use, and actigraphy-measured physical activity were collected.

Psychotic symptoms were screened by the Positive and Negative Syndrome Scale Remission tool (PANSS-r), that includes eight core symptoms of schizophrenia: general psychopathology (2 items), positive symptoms (3 items) and negative symptoms (3 items), scored from 1 (absent) to 7 (extreme) (Kay et al., 1987; Van Os et al., 2006).

Psychosocial functioning was assessed using the Health of the Nation Outcome Scales (HoNOS) or HoNOS 65+ for elderly people (Mulder et al., 2004; Wing et al., 1998). Both scales consist of 12 items, divided into four subscales (behavioral, symptomatic and social problems and impairment), scored from 0 (no problem) to 4 (very severe problem).

Quality of life was scored on the EuroQol-5D (EuroQol Group, 2011) and the brief World Health Organization Quality of Life Assessment scale (WHOQoLBref) (De Vries & Van Heck, 1997; Su et al., 2014). The EQ-5D consists of five items, each measuring a dimension of health: mobility, self-care, usual activities, pain/discomfort and anxiety/depression, rated from 1 (no problems) to 3 (many problems). We calculated an index score ranging from 0 (worst QoL) to 1 (perfect QoL) (Lamers et al., 2006). The WHOQoL-Bref contains 24 items that represent four domains of one's perceived quality of life: the physical (7 items), psychological (6 items), social (3 items), and environmental domain (8 items). Item scores ranged from 1 (very dissatisfied) to 5 (very satisfied) and were transformed into domain scores ranging from 4 to 20, according to the WHO guidelines (WHO, 1996).

The following physical health parameters were assessed: weight, abdominal girth, blood pressure, fasting glucose, triglycerides, total and HDL-cholesterol. Mean arterial pressure was calculated as the sum of one-third systolic blood pressure and two-thirds diastolic blood pressure. Psychotropic and somatic medication use was converted into defined daily dose (DDD) according to the Anatomical Therapeutic Chemical (ATC) Classification System (World Health Organisation, 2017).

Physical activity was measured with the ActiGraph GT3X+ (ActiGraph, Pensacola, Florida, VS), a hip-worn triaxial accelerometer. For the current analysis, average total activity counts per hour (TAC/h) was used as a continuous and detailed outcome variable of physical activity during daytime, where more counts indicate a higher level of physical activity.

Severity of psychosocial functioning was scored by the responsible psychiatrist or nurse practitioner (not blinded to the treatment condition) and all other data were collected by trained research assistants. Although research assistants were not actively informed about the treatment condition, blinding was not assured due to visible differences in the day-to-day program. A detailed description of used settings and criteria for valid measurement is described elsewhere (Kruisdijk et al., 2017).

### Statistical Analyses

#### Descriptives and Pre-processing of Data

All statistical tests were conducted using R version 4.04 (R Team, 2013). First, baseline characteristics were compared between groups (MULTI vs. TAU) using Chi-squared statistics for categorical variables and independent t-tests for continuous variables. Change scores of the variables from baseline to 18-month follow-up were calculated. Independent t-tests were performed to determine whether change scores differed between the two treatment conditions, using Bonferroni corrections for statistical significance (i.e., p<.0045, based on 11 comparisons). Continuous variables were examined for normality and homogeneity by comparing means with medians and standard deviations and by analyzing frequency histograms and normality plots. Second, we inspected the data for missing values of the selected variables. Data was missing in 0.94-9.4% of the variables, resulting in 90 complete and 16 incomplete cases. Missing data was inspected by groups of complete and incomplete cases using Chi-squared or independent t-tests. Missing data was assumed to be missing at random and was imputed using multiple imputation with chained equations, using the *MICE* package (van Buuren & Groothuis-Oudshoorn, 2011). Predictive mean matching was used to impute missing values, the preferred approach for multiple imputation that produces the least biased estimates (Marshall et al., 2010; van Buuren & Groothuis-Oudshoorn, 2011). We imputed the data five times and randomly selected one imputed dataset to estimate our network model. To ensure that our findings were not influenced by the selected the imputed dataset, we also estimated a network on the four other imputed datasets and checked if the most important edges were included in all networks, guided by previous literature (see Supplement) (Liu et al., 2021).

#### Network Construction

We used NIA to examine the impact of MULTI on the changes in physical and mental health outcomes. For the NIA, we included the change scores from baseline to follow-up of the outcome measures as continuous variables, and the treatment allocation (MULTI or TAU) as a binary variable. Considering the relatively small sample size (n=106) for network analysis, we used a selection of 11 variables representing the symptomatic, functional and physical health outcome domains measured in the sample, considering the most significant changes in previous research (Deenik et al., 2018a, 2018b, 2019) and most relevant outcome measures. Selected variables were: sum scores of the positive and negative symptoms (PANSS-r), social functioning (HoNOS), quality of life (EQ-5D and WHOQOL-BREF), psychotropic and somatic medication (DDD), total physical activity (TAC/h), abdominal girth, total cholesterol, and mean arterial pressure. Also, we added two baseline measures that differed significantly between the MULTI and TAU group: symptom severity (CGI; mean difference -0.79, 95% CI -1.23 to -0.29) and age (mean difference 6.51, 95% CI 2.05 to 10.96). Diagnosis did also differ between groups (χ^2^(1)=15.83, p<.0001), but was significantly (t(31)=2.73, p=.01) related to symptom severity, and was therefore not added to the model. The continuous variables included in the network model were normally distributed and could therefore be included for the network analysis without any transformation procedures.

A Mixed Graphical Model (MGM) was used to estimate the network using the *bootnet* (Epskamp et al., 2018) and *mgm* (Haslbeck & Waldorp, 2020) package. Networks are composed of nodes (variables) and edges, where the edges represent undirected conditional dependence relationships between the nodes. Thus, they indicate the association between two nodes controlling for their associations with all other variables of the network. We applied LASSO regularization to estimate the network structure and because sample size was relatively small for the number of parameters, we applied cross-validation to select the LASSO tuning parameter, a recommended method to discover the most important edges and the overall network structure in small samples (Isvoranu & Epskamp, 2021). Following recommendations in this field, we used nonparametric bootstrapping (bootstrapped samples n=1000) to assess accuracy of the edge estimates (see Supplement) (Epskamp et al., 2018).

#### Sensitivity Analyses

We have run sensitivity analyses to assess to what extent the included links were sensitive to i) imputation of missing data, ii) tuning parameter selection and iii) inclusion of baseline variables (CGI and age). We inspected the networks visually and we compared *node strength* between the networks, using the *qgraph* package (Epskamp et al., 2012). Node strength is a centrality index that quantifies how strong a node is indirectly connected to other nodes. Results can be found in the Supplement.

## Results

### Sample

**Fig. 1:**
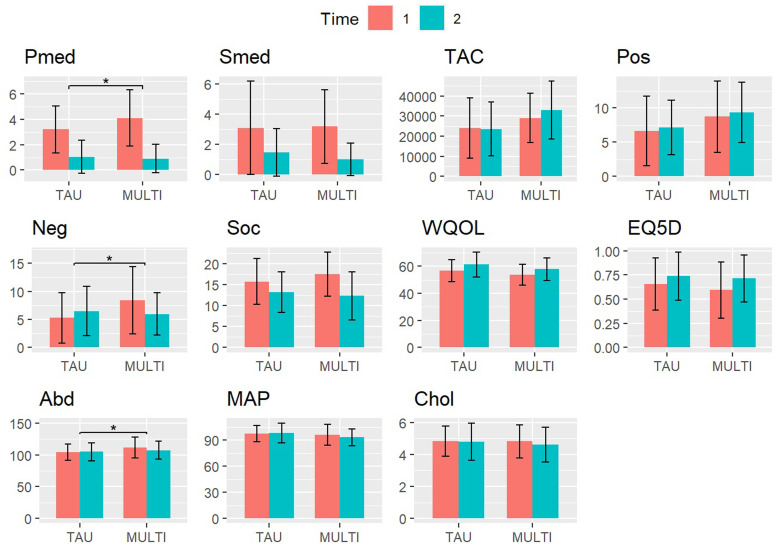
Scores of physical and mental health-related outcomes on baseline (T1) and 18-month follow-up (T2) in MULTI (n=65) and TAU (n=41). Significant differences between change scores are marked (* p<.0045, Bonferroni corrected). Note. Pmed, psychotropic medication; Smed, somatic medication; TAC, total activity counts (actigraphy measure for total physical activity); Pos, positive symptoms; Neg, negative symptoms; Soc, Health of the Nation Outcome Scale (higher scores indicate poorer social functioning); WQOL, WHO Quality of Life Brief Questionnaire; EQ5D, EuroQol 5D; Abd, abdominal girth; MAP, mean arterial pressure; Chol, total cholesterol.

The sample consisted of 106 subjects (MULTI, n=65; TAU, n=41), of whom 66 (62%) were male and 40 (38%) were female. Mean age was 54.7 years (SD=10.8) and 82.1% of patients were diagnosed with schizophrenia or other psychotic disorder, 17.9% with another diagnosis (e.g., bipolar disorder, personality disorder). Measurements at baseline and follow-up of the included physical and mental health-variables are presented in Table 1 and Fig. 1. Differences in change scores from baseline to follow-up were statistically significant (p<.0045) between groups for psychotropic medication (mean difference 1.04, 95% CI 0.36 to 1.73), negative symptoms (mean difference 3.76, 95% CI 1.59 to 5.93), and abdominal girth (mean difference 4.57, 95% CI 1.51 to 7.64).

**Table 1:** Demographic and clinical characteristics of the sample (n=106) on baseline and 18-month follow-up.

	TAU (N = 41)				MULTI (N = 65)			
	Baseline		Follow-up		Baseline		Follow-up	
Age, mean (SD)	58.7	(12.4)			52.2	(8.9)		
Sex, No. (%) male	23	(66)			43	(54)		
DSM-IV Diagnosis, No. (%)								
Schizophrenia or other psychotic disorder	26	(63)			61	(94)		
Other	15^1^	(37)			4^2^	(6)		
CGI, mean (SD)	4.20	(1.2)			4.95	(1.2)		
DDD psychotropic medication, mean (SD)	3.19	(1.8)	1.04	(1.3)	4.10	(2.2)	0.91	(1.1)
DDD somatic medication, mean (SD)	3.09	(3.1)	1.44	(1.6)	3.18	(2.5)	0.99	(1.1)
Total activity counts / hour, mean (SD)	24,025	(14,931)	23,574	(13,466)	29,102	(12,371)	33,021	(14,453)
PANSS positive symptoms, mean (SD)	6.6	(5.1)	7.1	(4.0)	8.7	(5.2)	9.3	(4.4)
PANSS negative symptoms, mean (SD)	5.3	(4.5)	6.5	(4.4)	8.4	(6.0)	6.0	(3.8)
HoNOS, mean (SD)	15.8	(5.5)	13.2	(4.9)	17.5	(5.3)	12.3	(5.8)
WHOQOL-BREF, mean (SD)	56.7	(8.0)	61.4	(9.2)	53.8	(7.7)	58.0	(8.4)
EQ5D, mean (SD)	0.65	(0.27)	0.74	(0.25)	0.59	(0.29)	0.71	(0.24)
Abdominal girth (cm), mean (SD)	103.7	(12.7)	104.6	(14.4)	111.1	(16.5)	107.3	(14.2)
Mean arterial pressure (mmHg), mean (SD)	97.7	(9.6)	98.3	(11.6)	96.0	(12.0)	93.5	(9.7)
Total cholesterol (mmol/L), mean (SD)	4.83	(0.94)	4.79	(1.17)	4.81	(1.03)	4.60	(1.08)

*Note.* CGI, Clinical Global Impression Severity; DDD, Defined Daily Dose; EQ5D, EuroQol 5D; HoNOS, Health of the Nation Outcome Scale; MULTI, Multidisciplinary Lifestyle enhancing Treatment for Inpatients with Severe Mental Illness; PANSS, Positive and Negative Syndrome Scale Remission tool; WHOQOL-BREF, brief World Health Organization Quality of Life Assessment scale.

1 Pervasive disorder not otherwise specified (n=1), alcohol-related disorders (n=3), mood disorders (n=7), personality disorders (n=3), somatoform disorders (n=1). ^2^Pervasive disorder not otherwise specified (n=1), mood disorders (n=2), anxiety disorder (n=1).

### Network

**Fig. 2:**
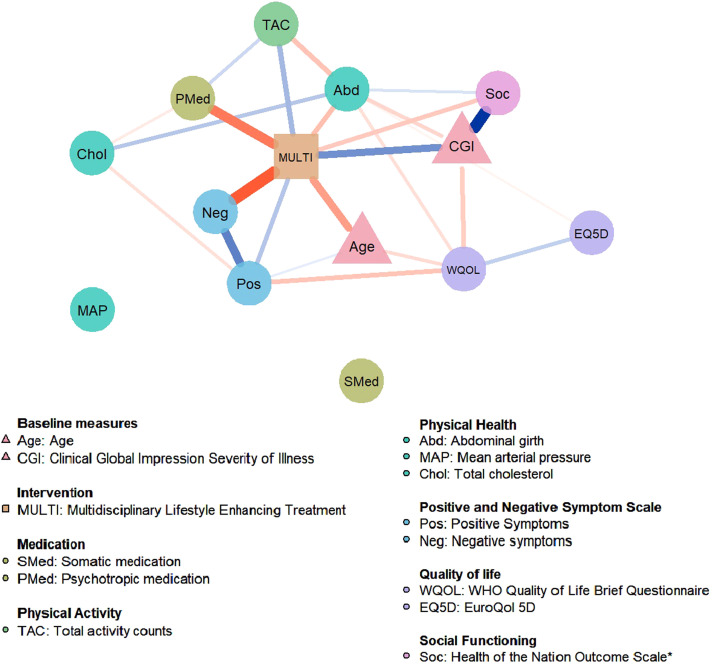
Estimated network for the multidisciplinary life-style intervention (MULTI) in relation to mental and physical health-related changes (n=106). Circular nodes represent change scores in the outcome variables, triangular nodes represent baseline variables, and the rectangular node represents the treatment allocation variable. Edges represent conditional dependence relationships between the variables; blue edges represent positive associations, whereas red edges represent negative associations. The thickness and saturation of the edge is proportional to the strength of the association. MULTI is directly related to a decrease of Neg, Pmed, Abd, Soc and an increase of TAC, and a slight increase of Pos. *Note.* *Higher scores on Soc indicate poorer social functioning.

Fig. 2 presents the network of MULTI in relation to mental and physical health-related changes from baseline to 18-month follow-up. In order of connection strength, results show that MULTI is directly connected to a decrease of negative symptoms (Neg), a decrease of psychotropic medication dosage (Pmed), an increase of actigraphy-measured physical activity (TAC), a decrease of abdominal girth (Abd), an increase of social functioning (reduction on the HoNOS score; Soc), and to an increase of positive symptoms (Pos). The network also identified associations between the different outcome variables, indicating that changes in these outcomes affect one another. These reciprocal connections were found within the outcome domains, for example increased scores within the quality of life (WQOL-EQ5D) and symptom severity (Pos-Neg) domain are related to each other. Also, connections were found between the different outcomes, for example increased positive symptoms (Pos) and decreased quality of life (WQOL) and increased physical activity (TAC) and decreased abdominal girth (Abd) are reciprocally related. Mean arterial blood pressure (MAP) and somatic medication use (Smed) were not connected to the network and visualized as separate nodes. The baseline variables CGI and Age are directly related to MULTI; Age was lower and CGI was higher in MULTI compared to TAU. Bootstrapping and sensitivity analysis showed that important direct links between MULTI and negative symptoms and MULTI and PMed were included in about 90% of the bootstrap samples, indicating high stability (see Supplement).

## Discussion

### Main Findings

Using NIA, this study examined the direct and indirect effects of a multidisciplinary lifestyle enhancing treatment on the physical and mental health-related outcomes in people with SMI. Our main finding was that MULTI was directly associated with improvements in a range of health-related outcomes (i.e., negative symptoms, social functioning, prescription of psychotropic medication, actigraphy-measured physical activity, and abdominal girth). Notably, these improvements in symptomatic, functional, and physical domains were independent of each other, suggesting a unique association between MULTI and changes in these distinct outcome domains. Secondly, we identified conditional associations between the other outcome measures within and between the different outcome domains, that may represent potential indirect effects of MULTI (e.g., MULTI is linked to a decrease in abdominal girth, which is in turn linked to a decrease in total cholesterol and may therefore indirectly influence cholesterol). To our knowledge, this is the first study that applied network estimation techniques to investigate direct and indirect effects of a lifestyle intervention in mental health research. The network model confirms the central role of MULTI in relation to independent changes in physical and mental health-related outcomes.

The network revealed that improvements in the different outcome variables were related in patterns, with connections that were often intuitively plausible. Only the direct link between MULTI and worsening of positive symptoms (e.g., hallucinations, paranoia, delusions, disorganization) could not be explained by clinical reasoning, as one would expect an absent or inverse relationship. When interpreting these findings, it is important to realize that the edges in the network represent conditional dependence relationships, meaning that they are conditioned for all other variables in the network. Considering the stronger links of MULTI-Neg and Neg-Pos, the positive relationship between MULTI-Pos could potentially be explained as a collider effect, resulting from a causal structure where the level of negative symptoms is caused both by MULTI and positive symptoms (i.e., MULTI → negative symptoms ← positive symptoms). That is, the treatment leads to a reduction in negative symptoms, and an increase in positive symptoms leads to an increase in negative symptoms. Conditioning on such a collider structure would explain the positive conditional dependence relationship between MULTI-Pos (Epskamp et al., 2022). This explanation is supported by our findings of i) a non-significant marginal association between MULTI-Pos and ii) an unstable edge between MULTI-Pos in the imputed datasets and bootstraps. Therefore, we are reluctant to interpret this positive link between MULTI and worsening of positive symptoms.

### Relevance of Findings

The results of this study shed light on the working mechanism of lifestyle interventions on physical and mental domains in people with SMI, by using novel network estimation techniques and thereby combining various subjectively and objectively measured health-related outcomes into one network model. Although previous studies have found similar effects of lifestyle interventions on physical activity interventions on cardiometabolic health, psychiatric symptom severity, and social functioning in people with SMI (Czosnek et al., 2019; Schmitt et al., 2018; Stubbs et al., 2018; Vancampfort et al., 2019), the underlying psychobiological mechanisms of these effects are still poorly understood (Stubbs et al., 2018). Separately analyzing health-related outcomes of lifestyle interventions, when they are interrelated, may impede the refinement of theory and thereby the development of effective, targeted interventions. By applying NIA, this study provides insight into the potential direct and indirect effects of a lifestyle intervention on both physical and mental outcome domains. These findings provide guidance for the development, research, and implementation of these interventions in routine daily care. Targeting of relevant outcome measures is a key element for the development of effective lifestyle intervention strategies and thus reducing excess mortality and burden of disease in people with SMI.

The treatment-induced changes in negative symptoms and social functioning in this study are particularly relevant since the search for interventions targeting negative symptoms and cognitive dysfunction is a major issue in schizophrenia research. Antipsychotics are effective to reduce positive symptoms, but are generally of less benefit for negative symptoms and cognitive deficits. Other psychosocial and psychological interventions (e.g., cognitive behavioral therapy, assertive community treatment) may reduce negative and cognitive symptoms and improve social functioning (Bighelli et al., 2021; Vita et al., 2021), but they are costly and access is poor (Schizophrenia Commission, 2012). The current literature supports the findings of this study, which demonstrates the effect of lifestyle interventions on improving negative symptoms, cognitive deficits, and psychosocial functioning in individuals with SMI (Firth et al., 2020). Therefore, lifestyle interventions could serve as effective, accessible, and low-cost treatment options to improve disease outcome in SMI.

### Limitations

Some limitations of this study should be considered. The first and most important limitation is the sample size of the study that is relatively small for a network analysis, which limits the generalizability and stability of the results (Isvoranu & Epskamp, 2021). For this reason, we conducted an exploratory analysis to identify the most important edges and overall network structure and performed additional analyses (i.e., bootstrapping and sensitivity analyses) to examine the accuracy and stability of the network. Furthermore, we included a limited number of variables in the network model to account for the relatively small sample size. By using previous literature (Czosnek et al., 2019; Schmitt et al., 2018; Stubbs et al., 2018; Vancampfort et al., 2019) and previously analyzed results from this study (Deenik et al., 2018a, 2018b, 2019), we aimed to select the most clinically relevant outcome variables. Although results need to be replicated in larger samples, this study is the first to explore direct and indirect effects of a lifestyle intervention on various physical and psychological outcome domains in patients with severe mental illness and may therefore provide important leads for future research (Isvoranu & Epskamp, 2021).

Second, as we estimated undirected conditional dependence relationships in the network model, causal relationships cannot be implied. In addition, we could not investigate the temporal development of changes in the outcome measures since we only had pre- and post-treatment measurements. Still, results from this study may be helpful in generating hypotheses regarding the working mechanisms of treatment by identifying direct and indirect lifestyle-intervention-related changes in the outcome measures.

Third, due to the observational nature of this study, whereby MULTI was already implemented pragmatically in three wards before start of the study, no randomization took place. Consequently, the MULTI and TAU groups were not similar in size and characteristics, which may have confounded our results. Therefore, we accounted for baseline differences in our analysis, by including the baseline variables that differed between groups as nodes in the network model and comparing the networks with and without the baseline variables (see Supplement) .

Limitations should be considered in future studies by using larger sample sizes, including multiple time points, and by adding more outcome variables (e.g., cognitive outcome measures) or by breaking variables down into subgroups (e.g., subgroups of psychotropic medication) to investigate lifestyle-intervention-related changes into more detail.

### Conclusion

This study provides a novel network approach to unravelling the complex effects of lifestyle interventions on physical and mental health outcomes in patients with SMI. Findings indicate that a multidisciplinary lifestyle intervention may directly influence (negative) symptom severity, medication use, social functioning, and physical activity. These insights provide guidance for the development, research, and implementation of lifestyle intervention strategies in people with SMI to improve their poor health status and reduced life expectancy.

## Declaration of competing interest

The authors declare that they have no known competing financial interests or personal relationships that could have appeared to influence the work reported in this paper.
